# Hydrogen Peroxide Inhalation During Dental Whitening Leading to Massive Hemoptysis and Tumor-Like Endobronchial Obstruction: A Case Report

**DOI:** 10.7759/cureus.102009

**Published:** 2026-01-21

**Authors:** Redouene Sid Ahmed Benazzouz, Massinissa Benyagoub, Nassim Nedjar

**Affiliations:** 1 Pulmonology Department, Laghouat Public Hospital, Laghouat, DZA; 2 Forensic Medicine Department, Laghouat Public Hospital, Laghouat, DZA

**Keywords:** bronchial obstruction, dental whitening, hemoptysis, hydrogen peroxide inhalation, pulmonary atelectasis, tooth bleaching

## Abstract

Hydrogen peroxide (H₂O₂) is widely used in dental whitening and is generally considered safe when applied under professional supervision. However, accidental aspiration may cause unexpected airway complications.

A 55-year-old North African woman presented with massive hemoptysis, preceded by a 3-month history of persistent dry cough and an earlier minor hemoptysis episode. Symptoms had begun shortly after a dental whitening procedure using 35% H₂O₂, complicated by intense pharyngeal pain, ulcerative lesions, and severe coughing. Chest imaging showed complete left lower lobe atelectasis due to an intraluminal soft-tissue lesion, with associated parenchymal consolidation. Flexible bronchoscopy was attempted but interrupted due to complications, after which the patient expectorated thick mucus. Follow-up imaging one month later demonstrated complete resolution of the lesion and full lung re-expansion.

This case highlights the potential for severe airway injury following dental use of high-concentration H₂O₂. Awareness of such iatrogenic exposures is essential when evaluating unexplained hemoptysis and atelectasis.

## Introduction

Hydrogen peroxide (H₂O₂) is commonly used in daily medical and dental practice, particularly for surface disinfection and tooth whitening. In dental bleaching, low concentrations ranging from 3% to 10% are typically used for at-home products, whereas in-office procedures applied under professional supervision often rely on higher concentrations of 25-40% H₂O₂. In contrast, hydrogen peroxide solutions used for household disinfection usually contain around 3%, while medical disinfectants may range from 3% to 6% in routine use [1]. While generally safe when applied under controlled conditions, exposure of the respiratory tract to concentrated solutions or vapors can result in chemical injury. Experimental data show that inhaled H₂O₂ induces oxidative stress, ciliary dysfunction, and epithelial cell damage [2]. In clinical settings, accidental inhalation has been linked to upper airway irritation and to chemical pneumonitis or pulmonary edema. Reported cases have generally involved non-controlled aerosolized exposures rather than regulated clinical use [3].

Endobronchial lesions secondary to H₂O₂ exposure, however, are exceedingly rare. In previously published cases of hydrogen peroxide inhalation, bronchoscopy findings are rarely detailed, as most patients are managed early for acute respiratory symptoms, making the assessment of focal bronchial lesions uncommon. This is clinically important because cough, hemoptysis, and lobar collapse are common presenting features of bronchogenic carcinoma, and such cases may therefore create significant diagnostic uncertainty.

The purpose of this report is to describe an unusual case of massive hemoptysis and a pseudotumoral endobronchial lesion following dental whitening with 35% H₂O₂. A PubMed search using the terms ‘hydrogen peroxide’ combined with ‘inhalation’, ‘aspiration’, ‘tooth bleaching’, ‘dental whitening’, ‘bronchus’, ‘endobronchial’, and ‘airway’ did not identify any previous reports of a pseudotumoral endobronchial obstruction after hydrogen peroxide exposure. To our knowledge, this case therefore expands the clinical spectrum of H₂O₂-induced pulmonary injury and emphasizes the importance of including chemical airway injury in the differential diagnosis of endobronchial masses.

This case suggests that concentrated hydrogen peroxide, used in routine dental whitening, may cause severe airway injury mimicking bronchogenic carcinoma. The patient developed massive hemoptysis, a pseudotumoral endobronchial lesion, and lobar atelectasis, which resolved after conservative management. Awareness of this potential complication may improve diagnostic reasoning when evaluating unexplained hemoptysis and lobar atelectasis, and help clinicians consider chemical airway injury among the possible mechanisms rather than malignancy alone.

## Case presentation

A 55-year-old North African woman, employed as a cook using open-fire stoves, was admitted for evaluation of massive hemoptysis. She was a lifelong non-smoker with no history of chronic respiratory or cardiovascular disease, coagulation disorder, or relevant family history, and no known exposure to industrial or chemical fumes. She had no known genetic conditions or prior major interventions.

She reported a three-month history of persistent dry cough, initially associated with a minor hemoptysis episode and later complicated by non-hypoxemic dyspnea at rest. During this period, she did not undergo any formal medical evaluation. She lived in a remote area with limited access to specialist care and managed her symptoms at home with over-the-counter remedies. The clinical course then culminated in a massive hemoptysis episode, prompting emergency admission.

Around the onset of her symptoms, the patient had undergone a dental whitening procedure using 35% hydrogen peroxide, complicated by severe pharyngeal pain, oral ulcerations, and intense coughing. This exposure was not disclosed to the treating team during the initial hospitalization and was only mentioned retrospectively during follow-up.

On admission, physical examination revealed diminished breath sounds over the left hemithorax, with no cyanosis, clubbing, or lymphadenopathy. Oral inspection did not reveal any active bleeding source or visible mucosal lesion and showed complete edentulism of the upper jaw and absence of molars and premolars on the lower jaw.

Initial laboratory tests showed a normal complete blood count, including white blood cell count, and no elevation of C-reactive protein or other inflammatory markers. Coagulation profile, renal function tests, and liver enzymes were within normal ranges.

**Figure 1:**
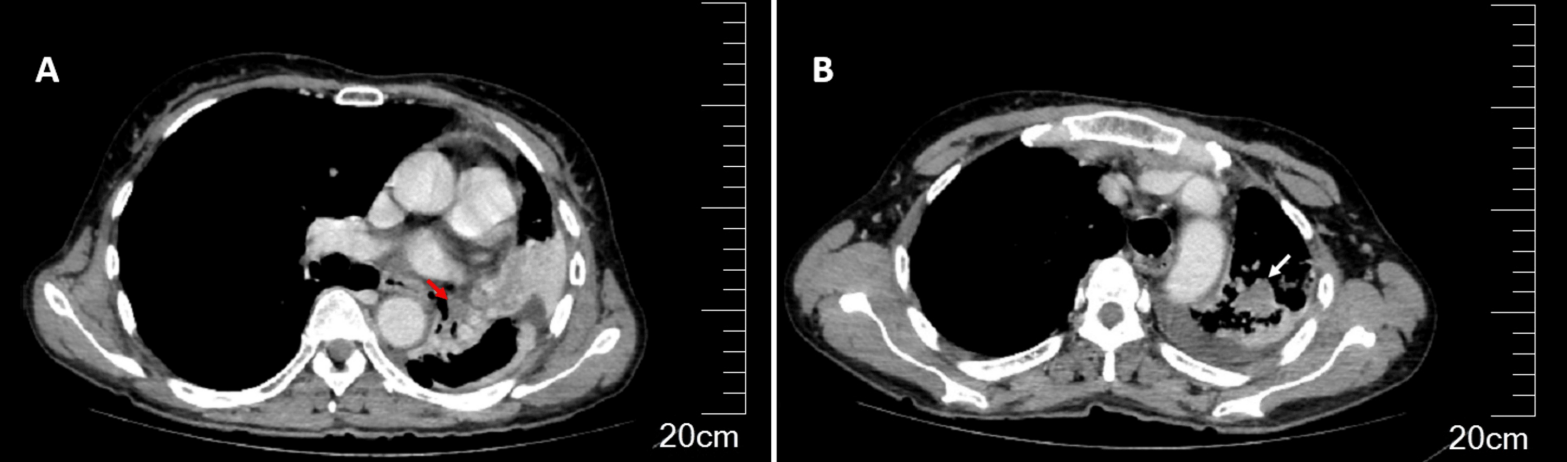
Imaging at presentation: endobronchial obstruction mimicking malignancy Axial chest CT images (a, b) demonstrating an intraluminal hypo-enhancing soft-tissue density lesion within the left lower lobar bronchus (red arrow), with a complete collapse of the left lower lobe. A separate posterior heterogeneous hypoattenuating left upper lobe parenchymal consolidation is also observed (white arrow), with a small associated pleural effusion.

Chest radiography demonstrated a mediastinal shift toward the left side and elevation of the left hemidiaphragm, findings consistent with significant left-sided atelectasis. Chest CT revealed an intraluminal soft-tissue-density hypo-enhancing lesion obstructing the left lower lobar bronchus, leading to complete collapse of the left lower lobe, with an additional posterior left upper-lobe heterogeneous consolidation and mild pleural effusion (Figure 1).

A contrast-enhanced CT pulmonary angiography did not demonstrate any pulmonary embolism, vascular malformation, or focal vascular lesion that could account for the bleeding. Repeated sputum tests for *Mycobacterium tuberculosis* were negative.

Flexible bronchoscopy was attempted but had to be interrupted due to periprocedural hypoxemia, persistent cough, and hypertensive crisis. Before the procedure was aborted, the left upper lobar bronchus was seen to be patent, without visible mucosal abnormalities, whereas the entrance of the left lower lobar bronchus was obscured by abundant thick mucus plugging the lumen, with no clearly identifiable solid mass or active bleeding source. Shortly after the procedure, the patient expectorated a large quantity of thick mucus, followed by gradual clinical improvement. Bacteriological analysis of the thick secretions expectorated after the bronchoscopy, including specific testing for mycobacteria, did not identify *Mycobacterium tuberculosis,* and routine cultures yielded no pathogenic bacterial growth.

After the endoscopy, the patient received etamsylate as hemostatic therapy and was kept under clinical and hemodynamic monitoring. As she remained stable and free of recurrent bleeding, she was discharged the following day with no specific medication and a recommendation for repeat bronchoscopy under general anesthesia in a referral center. However, as her recovery over the following months was uneventful and she declined further invasive procedures, no interval bronchoscopy was performed. Four months after symptom onset, follow-up chest CT demonstrated complete resolution of the endobronchial lesion and full re-expansion of the left lung, with only minor reticulations suggestive of sequelae. The patient remained asymptomatic, with no recurrence of cough or hemoptysis.

## Discussion

The retrospective identification of exposure to 35% hydrogen peroxide during a dental whitening procedure provided a plausible mechanism for the patient’s airway injury. Although the physiopathological plausibility is strongly suggestive, a direct causal link cannot be definitively established, and alternative explanations, such as a non-specific post-hemorrhagic obstructive process or an undetected benign endobronchial lesion, cannot be completely ruled out. The acute pharyngeal pain, ulceration, and severe coughing suggest mucosal irritation, which we hypothesize to be related to inhalation of vapors or micro-aspiration of the whitening solution. However, the exact route and extent of exposure remain inferred rather than proven. We propose that this sequence may explain the subsequent pseudotumoral endobronchial obstruction, lobar collapse, and hemoptysis observed.

At presentation, the three-month history of cough, hemoptysis, and imaging showing an irregular endobronchial lesion with lobar collapse strongly mimicked bronchogenic carcinoma. Endobronchial tuberculosis, which may also cause chronic cough, hemoptysis, and obstructive atelectasis, was considered but ruled out by negative microbiology and full spontaneous resolution. The dramatic expectoration post-bronchoscopy is best explained by the expulsion of obstructive bronchial material - mucus, inflammatory debris, or clot - which had mimicked a tumor radiologically. This mechanism, likely promoted by hydrogen peroxide-induced airway injury, accounts for both the pseudotumoral appearance and its spontaneous resolution [4]. However, no histological or cytological examination of the expelled material was performed, which limits diagnostic certainty.

Hydrogen peroxide’s oxidant properties are well-documented. Controlled human exposure studies at very low concentrations (0.5-2.2 ppm) have shown only mild, transient upper-airway irritation without significant pulmonary dysfunction [5]. In vitro, it impairs ciliary motility and promotes epithelial cell death [6]. However, accidental inhalation of aerosolized high-concentration solutions has been associated with clinically significant chemical pneumonitis [3]. In this patient, exposure to high-concentration peroxide probably induced acute airway injury, inflammation, and viscous secretions acting as an obstructive mass.

This case, therefore, illustrates an unusual but plausible mechanism of reversible, peroxide-induced endobronchial obstruction mimicking malignancy. It highlights three practical lessons: systematic consideration of chemical exposures in atypical airway disease, recognition that sudden expectoration may reveal a reversible obstructive lesion, and the need for caution in the cosmetic use of high-concentration oxidants in dental practice [1]. These conclusions, however, must be interpreted in light of several limitations, including the absence of histologic or cytologic confirmation and the incomplete endoscopic visualization of the lesion. As with any single-case observation, alternative diagnoses therefore cannot be excluded with absolute certainty.

## Conclusions

This case suggests that severe airway complications may result from accidental inhalation or mucosal injury during dental whitening procedures involving high-concentration hydrogen peroxide. Clinicians should maintain a high index of suspicion for such exposures in patients presenting with unexplained airway obstruction, atelectasis, or hemoptysis, especially when imaging reveals a tumor-like endobronchial lesion mimicking malignancy. However, these conclusions must be interpreted in light of the limitations of this single-case report, including the absence of direct histologic confirmation of airway injury, the aborted bronchoscopy, and the inability to completely rule out all alternative causes.
